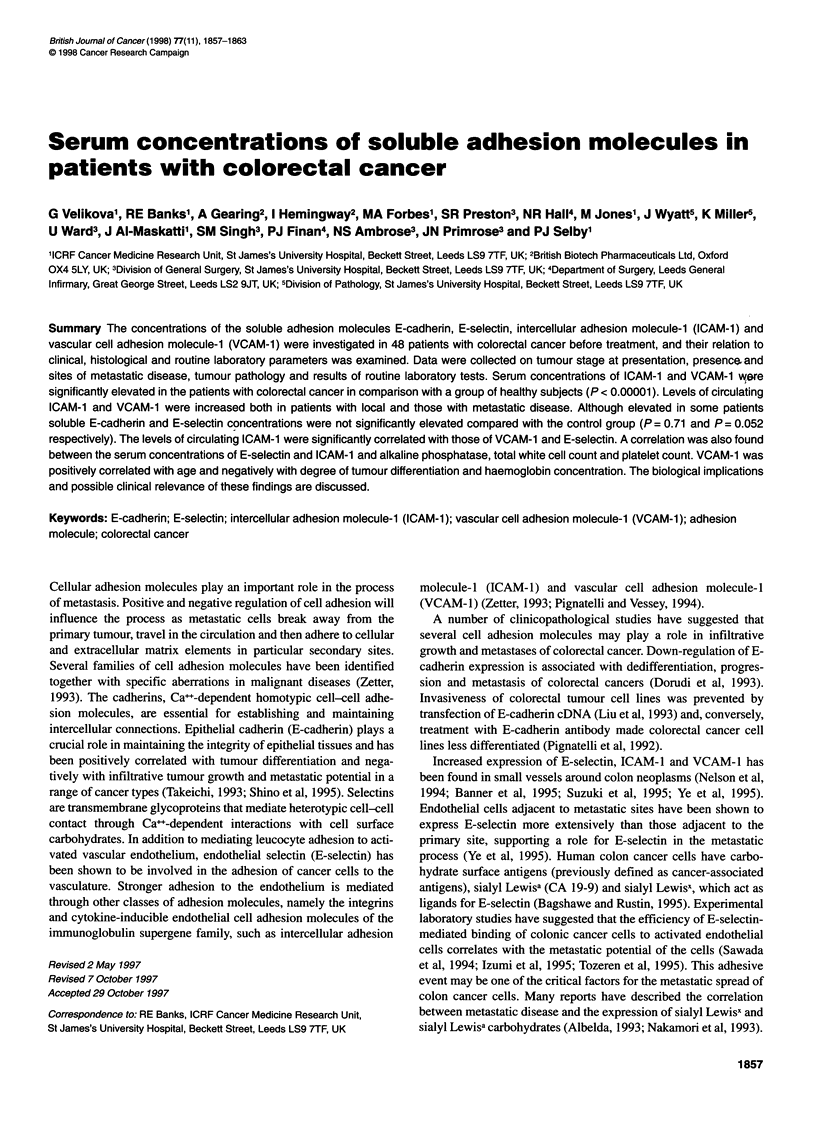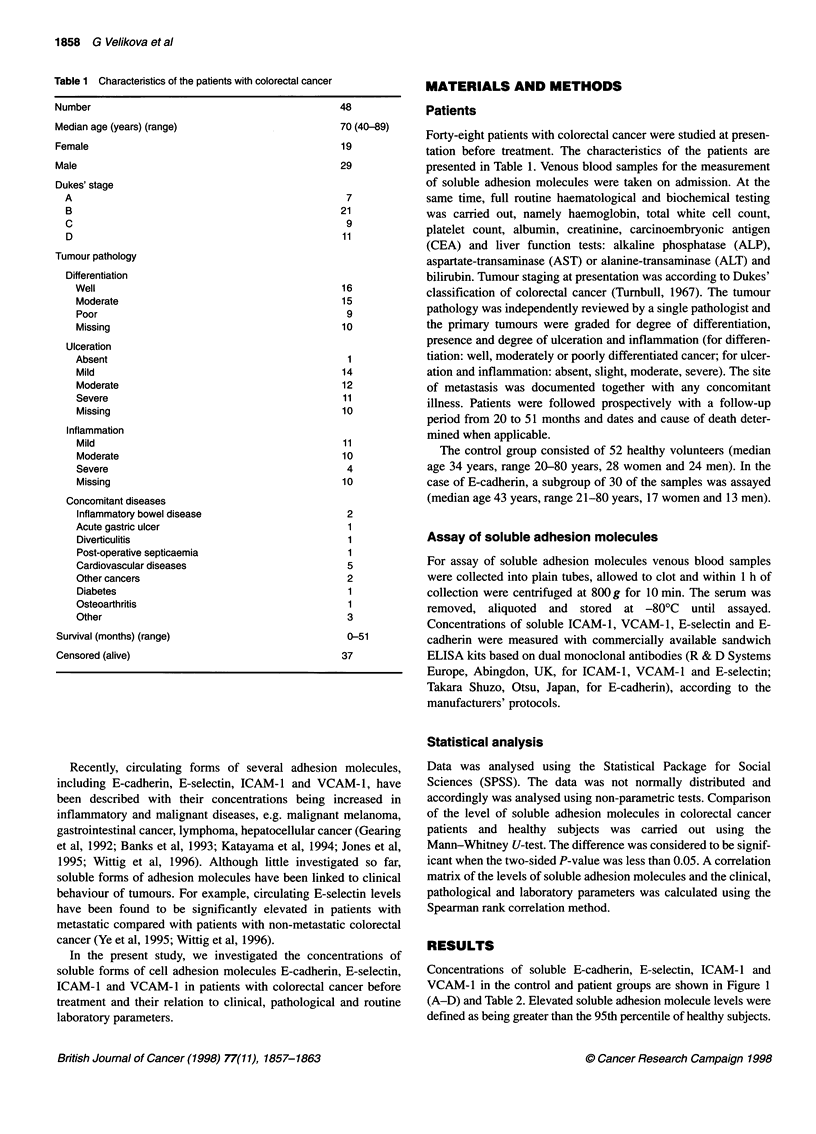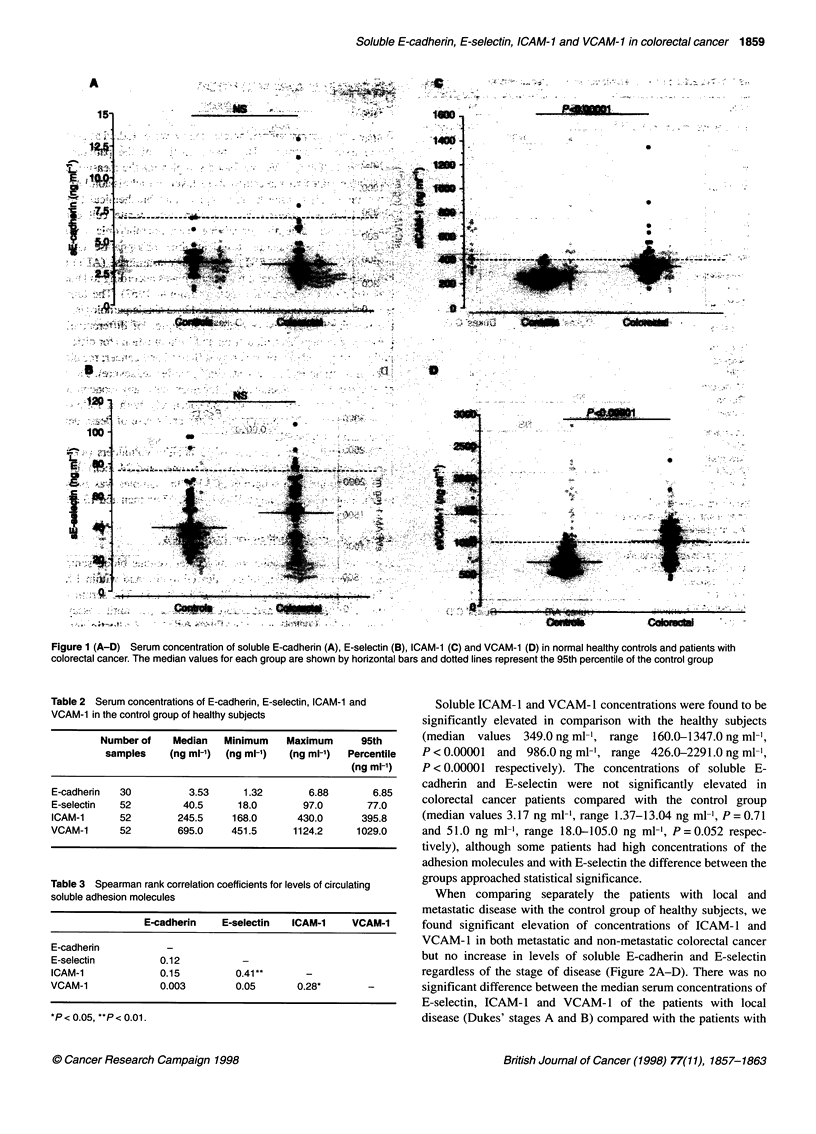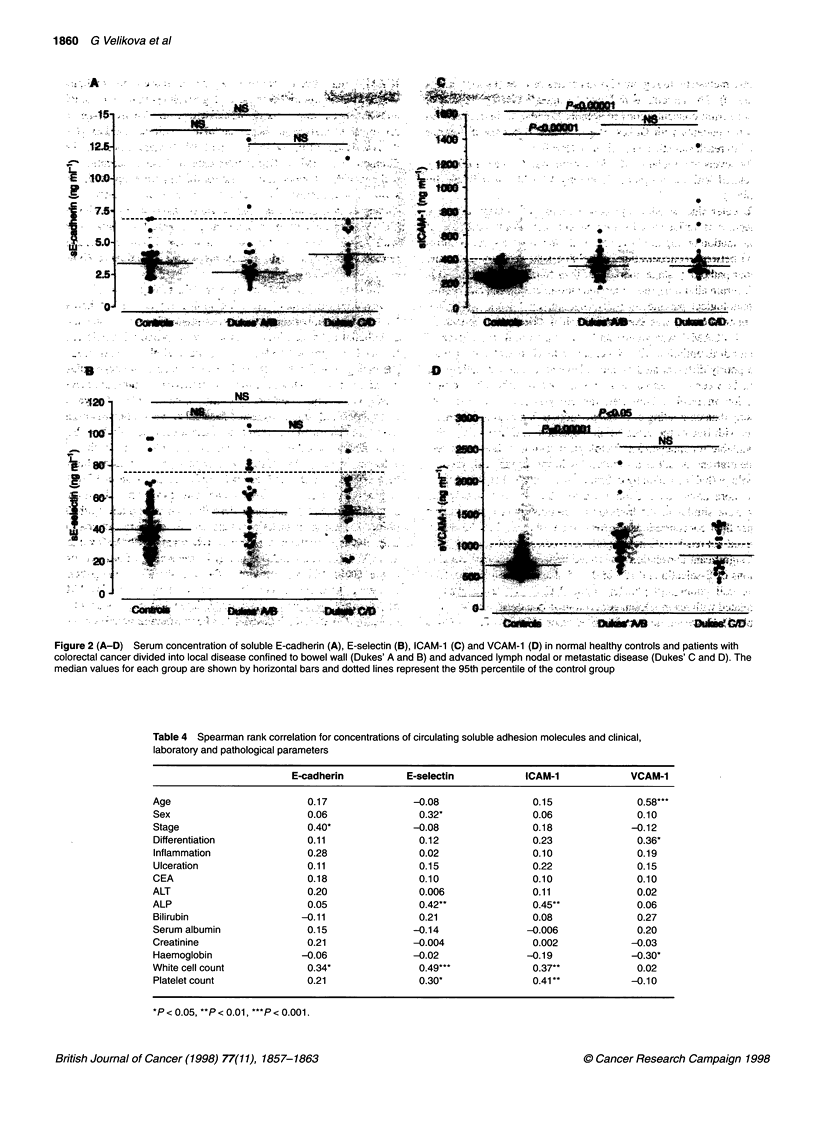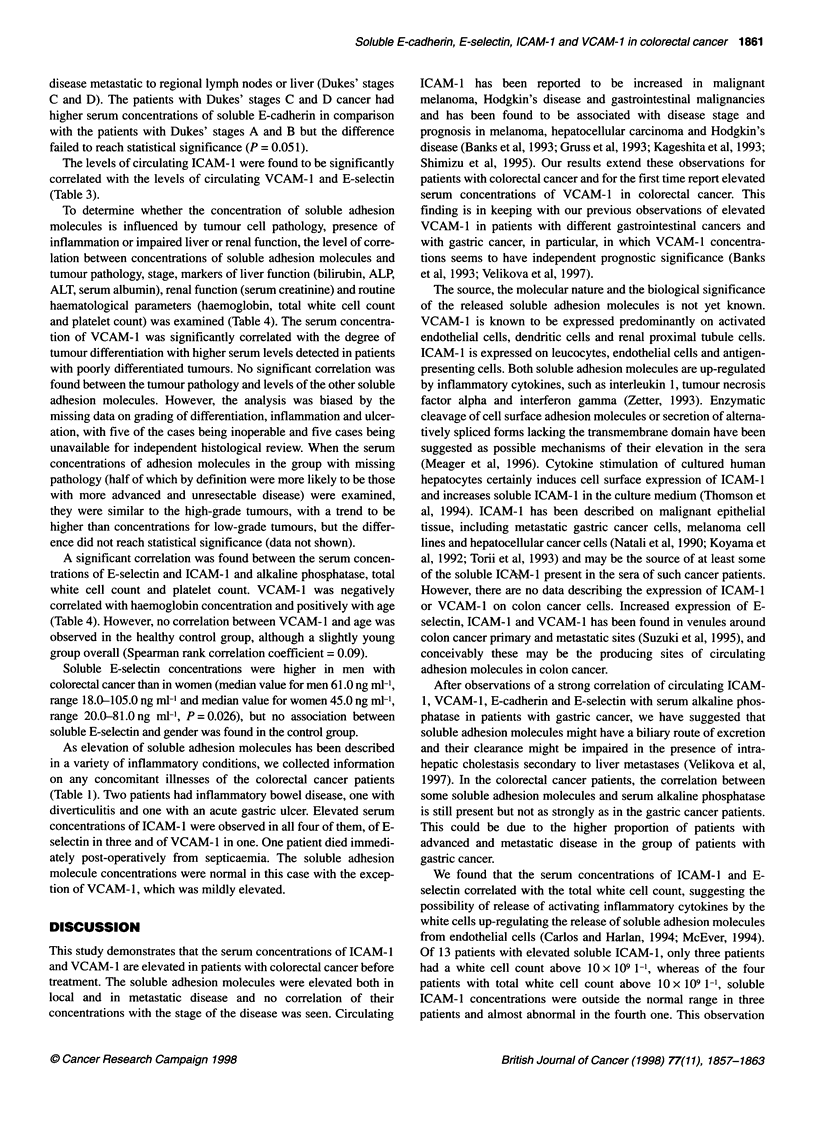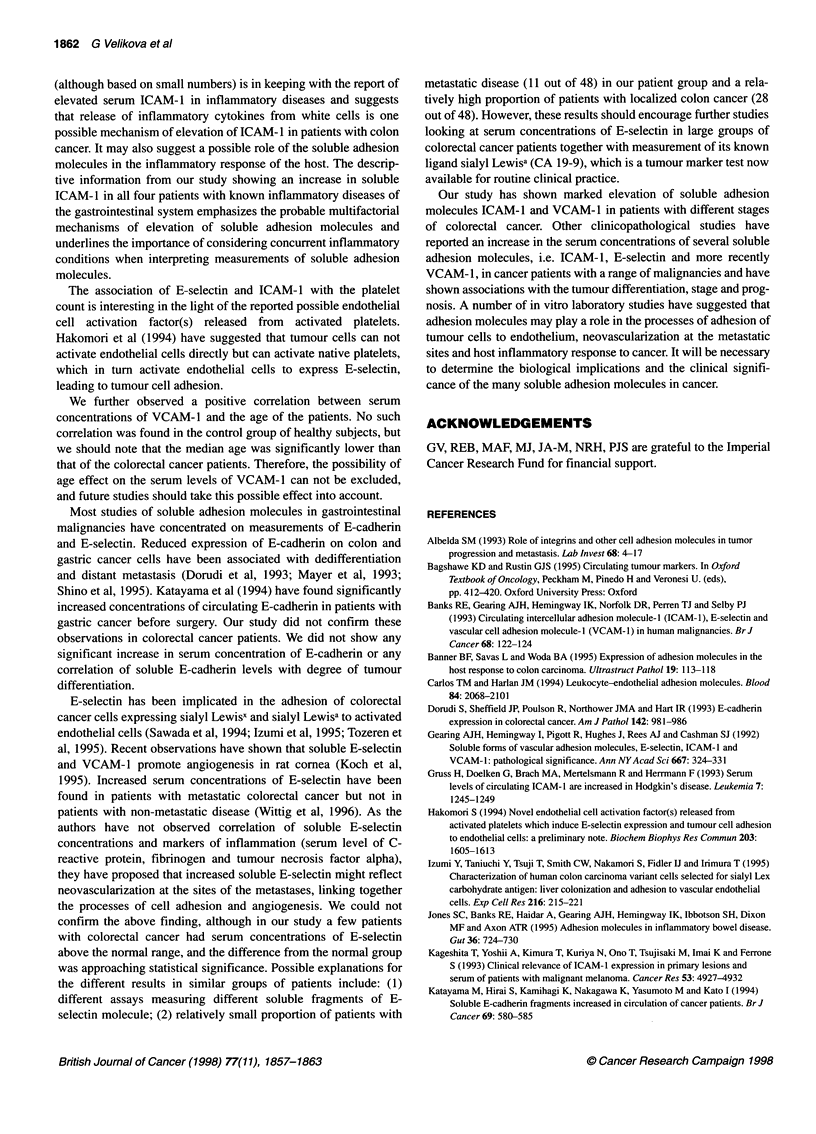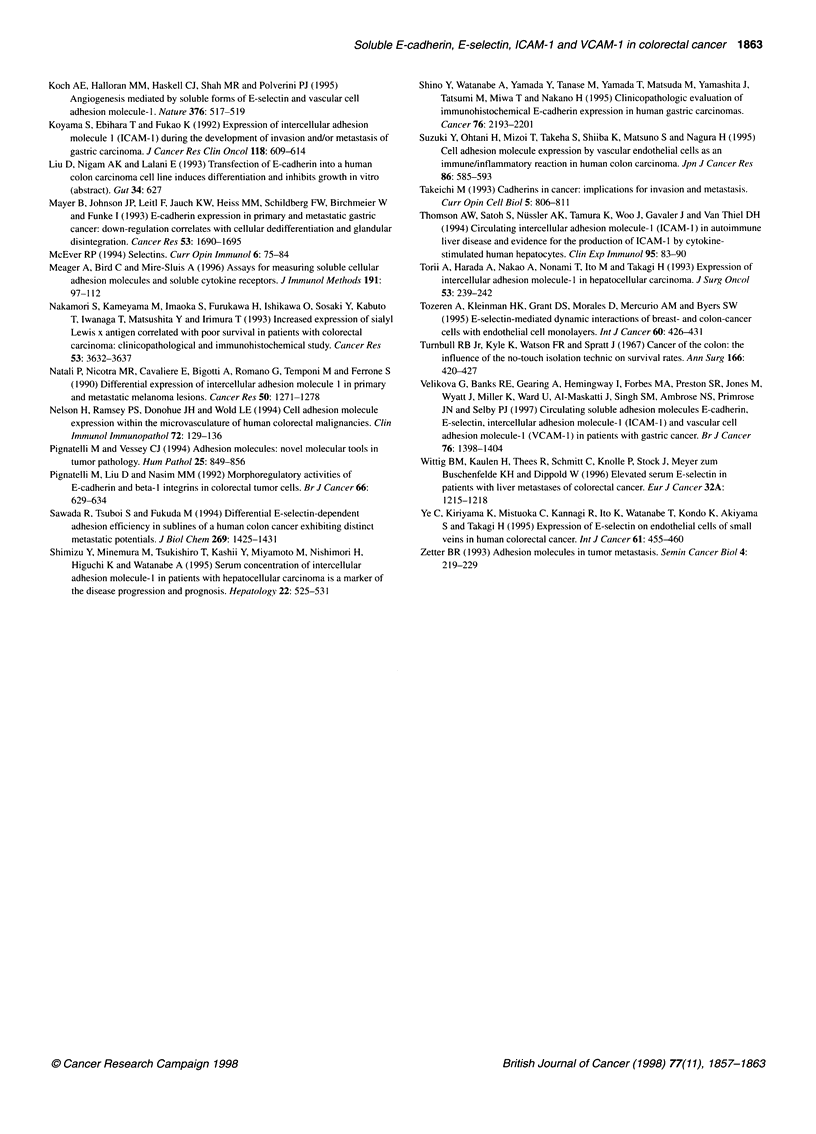# Serum concentrations of soluble adhesion molecules in patients with colorectal cancer.

**DOI:** 10.1038/bjc.1998.309

**Published:** 1998-06

**Authors:** G. Velikova, R. E. Banks, A. Gearing, I. Hemingway, M. A. Forbes, S. R. Preston, N. R. Hall, M. Jones, J. Wyatt, K. Miller, U. Ward, J. Al-Maskatti, S. M. Singh, P. J. Finan, N. S. Ambrose, J. N. Primrose, P. J. Selby

**Affiliations:** ICRF Cancer Medicine Research Unit, St James's University Hospital, Leeds, UK.

## Abstract

The concentrations of the soluble adhesion molecules E-cadherin, E-selectin, intercellular adhesion molecule-1 (ICAM-1) and vascular cell adhesion molecule-1 (VCAM-1) were investigated in 48 patients with colorectal cancer before treatment, and their relation to clinical, histological and routine laboratory parameters was examined. Data were collected on tumour stage at presentation, presence and sites of metastatic disease, tumour pathology and results of routine laboratory tests. Serum concentrations of ICAM-1 and VCAM-1 were significantly elevated in the patients with colorectal cancer in comparison with a group of healthy subjects (P < 0.00001). Levels of circulating ICAM-1 and VCAM-1 were increased both in patients with local and those with metastatic disease. Although elevated in some patients soluble E-cadherin and E-selectin concentrations were not significantly elevated compared with the control group (P = 0.71 and P = 0.052 respectively). The levels of circulating ICAM-1 were significantly correlated with those of VCAM-1 and E-selectin. A correlation was also found between the serum concentrations of E-selectin and ICAM-1 and alkaline phosphatase, total white cell count and platelet count. VCAM-1 was positively correlated with age and negatively with degree of tumour differentiation and haemoglobin concentration. The biological implications and possible clinical relevance of these findings are discussed.


British Journal of Cancer (1998) 77(11), 1857–1863
© 1998 Cancer Research Campaign

### Serum concentrations of soluble adhesion molecules in patients with colorectal cancer

G Velikova[1], RE Banks[1], A Gearing[2], I Hemingway[2], MA Forbes[1], SR Preston[3], NR Hall[4], M Jones[1], J Wyatt[5], K Miller[5], U Ward[3], J Al-Maskatti[1], SM Singh[3], PJ Finan[4], NS Ambrose[3], JN Primrose[3] and PJ Selby[1]

[1]ICRF Cancer Medicine Research Unit, St James's University Hospital, Beckett Street, Leeds LS9 7TF, UK; [2]British Biotech Pharmaceuticals Ltd, Oxford OX4 5LY, UK; [3]Division of General Surgery, St James's University Hospital, Beckett Street, Leeds LS9 7TF, UK; [4]Department of Surgery, Leeds General Infirmary, Great George Street, Leeds LS2 9JT, UK; [5]Division of Pathology, St James's University Hospital, Beckett Street, Leeds LS9 7TF, UK

**Summary** The concentrations of the soluble adhesion molecules E-cadherin, E-selectin, intercellular adhesion molecule-1 (ICAM-1) and vascular cell adhesion molecule-1 (VCAM-1) were investigated in 48 patients with colorectal cancer before treatment, and their relation to clinical, histological and routine laboratory parameters was examined. Data were collected on tumour stage at presentation, presence and sites of metastatic disease, tumour pathology and results of routine laboratory tests. Serum concentrations of ICAM-1 and VCAM-1 were significantly elevated in the patients with colorectal cancer in comparison with a group of healthy subjects ($P < 0.00001$). Levels of circulating ICAM-1 and VCAM-1 were increased both in patients with local and those with metastatic disease. Although elevated in some patients soluble E-cadherin and E-selectin concentrations were not significantly elevated compared with the control group ($P = 0.71$ and $P = 0.052$ respectively). The levels of circulating ICAM-1 were significantly correlated with those of VCAM-1 and E-selectin. A correlation was also found between the serum concentrations of E-selectin and ICAM-1 and alkaline phosphatase, total white cell count and platelet count. VCAM-1 was positively correlated with age and negatively with degree of tumour differentiation and haemoglobin concentration. The biological implications and possible clinical relevance of these findings are discussed.

**Keywords:** E-cadherin; E-selectin; intercellular adhesion molecule-1 (ICAM-1); vascular cell adhesion molecule-1 (VCAM-1); adhesion molecule; colorectal cancer

Cellular adhesion molecules play an important role in the process of metastasis. Positive and negative regulation of cell adhesion will influence the process as metastatic cells break away from the primary tumour, travel in the circulation and then adhere to cellular and extracellular matrix elements in particular secondary sites. Several families of cell adhesion molecules have been identified together with specific aberrations in malignant diseases (Zetter, 1993). The cadherins, $Ca^{++}$-dependent homotypic cell–cell adhesion molecules, are essential for establishing and maintaining intercellular connections. Epithelial cadherin (E-cadherin) plays a crucial role in maintaining the integrity of epithelial tissues and has been positively correlated with tumour differentiation and negatively with infiltrative tumour growth and metastatic potential in a range of cancer types (Takeichi, 1993; Shino et al, 1995). Selectins are transmembrane glycoproteins that mediate heterotypic cell–cell contact through $Ca^{++}$-dependent interactions with cell surface carbohydrates. In addition to mediating leucocyte adhesion to activated vascular endothelium, endothelial selectin (E-selectin) has been shown to be involved in the adhesion of cancer cells to the vasculature. Stronger adhesion to the endothelium is mediated through other classes of adhesion molecules, namely the integrins and cytokine-inducible endothelial cell adhesion molecules of the immunoglobulin supergene family, such as intercellular adhesion molecule-1 (ICAM-1) and vascular cell adhesion molecule-1 (VCAM-1) (Zetter, 1993; Pignatelli and Vessey, 1994).

A number of clinicopathological studies have suggested that several cell adhesion molecules may play a role in infiltrative growth and metastases of colorectal cancer. Down-regulation of E-cadherin expression is associated with dedifferentiation, progression and metastasis of colorectal cancers (Dorudi et al, 1993). Invasiveness of colorectal tumour cell lines was prevented by transfection of E-cadherin cDNA (Liu et al, 1993) and, conversely, treatment with E-cadherin antibody made colorectal cancer cell lines less differentiated (Pignatelli et al, 1992).

Increased expression of E-selectin, ICAM-1 and VCAM-1 has been found in small vessels around colon neoplasms (Nelson et al, 1994; Banner et al, 1995; Suzuki et al, 1995; Ye et al, 1995). Endothelial cells adjacent to metastatic sites have been shown to express E-selectin more extensively than those adjacent to the primary site, supporting a role for E-selectin in the metastatic process (Ye et al, 1995). Human colon cancer cells have carbohydrate surface antigens (previously defined as cancer-associated antigens), sialyl Lewis$^a$ (CA 19-9) and sialyl Lewis$^x$, which act as ligands for E-selectin (Bagshawe and Rustin, 1995). Experimental laboratory studies have suggested that the efficiency of E-selectin-mediated binding of colonic cancer cells to activated endothelial cells correlates with the metastatic potential of the cells (Sawada et al, 1994; Izumi et al, 1995; Tozeren et al, 1995). This adhesive event may be one of the critical factors for the metastatic spread of colon cancer cells. Many reports have described the correlation between metastatic disease and the expression of sialyl Lewis$^x$ and sialyl Lewis$^a$ carbohydrates (Albelda, 1993; Nakamori et al, 1993).

*Revised 2 May 1997*
*Revised 7 October 1997*
*Accepted 29 October 1997*

*Correspondence to:* RE Banks, ICRF Cancer Medicine Research Unit, St James's University Hospital, Beckett Street, Leeds LS9 7TF, UK

Table 1 Characteristics of the patients with colorectal cancer

| | |
|---|---|
| Number | 48 |
| Median age (years) (range) | 70 (40–89) |
| Female | 19 |
| Male | 29 |
| Dukes' stage | |
| A | 7 |
| B | 21 |
| C | 9 |
| D | 11 |
| Tumour pathology | |
| Differentiation | |
| Well | 16 |
| Moderate | 15 |
| Poor | 9 |
| Missing | 10 |
| Ulceration | |
| Absent | 1 |
| Mild | 14 |
| Moderate | 12 |
| Severe | 11 |
| Missing | 10 |
| Inflammation | |
| Mild | 11 |
| Moderate | 10 |
| Severe | 4 |
| Missing | 10 |
| Concomitant diseases | |
| Inflammatory bowel disease | 2 |
| Acute gastric ulcer | 1 |
| Diverticulitis | 1 |
| Post-operative septicaemia | 1 |
| Cardiovascular diseases | 5 |
| Other cancers | 2 |
| Diabetes | 1 |
| Osteoarthritis | 1 |
| Other | 3 |
| Survival (months) (range) | 0–51 |
| Censored (alive) | 37 |

Recently, circulating forms of several adhesion molecules, including E-cadherin, E-selectin, ICAM-1 and VCAM-1, have been described with their concentrations being increased in inflammatory and malignant diseases, e.g. malignant melanoma, gastrointestinal cancer, lymphoma, hepatocellular cancer (Gearing et al, 1992; Banks et al, 1993; Katayama et al, 1994; Jones et al, 1995; Wittig et al, 1996). Although little investigated so far, soluble forms of adhesion molecules have been linked to clinical behaviour of tumours. For example, circulating E-selectin levels have been found to be significantly elevated in patients with metastatic compared with patients with non-metastatic colorectal cancer (Ye et al, 1995; Wittig et al, 1996).

In the present study, we investigated the concentrations of soluble forms of cell adhesion molecules E-cadherin, E-selectin, ICAM-1 and VCAM-1 in patients with colorectal cancer before treatment and their relation to clinical, pathological and routine laboratory parameters.

### MATERIALS AND METHODS

### Patients

Forty-eight patients with colorectal cancer were studied at presentation before treatment. The characteristics of the patients are presented in Table 1. Venous blood samples for the measurement of soluble adhesion molecules were taken on admission. At the same time, full routine haematological and biochemical testing was carried out, namely haemoglobin, total white cell count, platelet count, albumin, creatinine, carcinoembryonic antigen (CEA) and liver function tests: alkaline phosphatase (ALP), aspartate-transaminase (AST) or alanine-transaminase (ALT) and bilirubin. Tumour staging at presentation was according to Dukes' classification of colorectal cancer (Turnbull, 1967). The tumour pathology was independently reviewed by a single pathologist and the primary tumours were graded for degree of differentiation, presence and degree of ulceration and inflammation (for differentiation: well, moderately or poorly differentiated cancer; for ulceration and inflammation: absent, slight, moderate, severe). The site of metastasis was documented together with any concomitant illness. Patients were followed prospectively with a follow-up period from 20 to 51 months and dates and cause of death determined when applicable.

The control group consisted of 52 healthy volunteers (median age 34 years, range 20–80 years, 28 women and 24 men). In the case of E-cadherin, a subgroup of 30 of the samples was assayed (median age 43 years, range 21–80 years, 17 women and 13 men).

### Assay of soluble adhesion molecules

For assay of soluble adhesion molecules venous blood samples were collected into plain tubes, allowed to clot and within 1 h of collection were centrifuged at 800 *g* for 10 min. The serum was removed, aliquoted and stored at −80°C until assayed. Concentrations of soluble ICAM-1, VCAM-1, E-selectin and E-cadherin were measured with commercially available sandwich ELISA kits based on dual monoclonal antibodies (R & D Systems Europe, Abingdon, UK, for ICAM-1, VCAM-1 and E-selectin; Takara Shuzo, Otsu, Japan, for E-cadherin), according to the manufacturers' protocols.

### Statistical analysis

Data was analysed using the Statistical Package for Social Sciences (SPSS). The data was not normally distributed and accordingly was analysed using non-parametric tests. Comparison of the level of soluble adhesion molecules in colorectal cancer patients and healthy subjects was carried out using the Mann–Whitney *U*-test. The difference was considered to be significant when the two-sided *P*-value was less than 0.05. A correlation matrix of the levels of soluble adhesion molecules and the clinical, pathological and laboratory parameters was calculated using the Spearman rank correlation method.

### RESULTS

Concentrations of soluble E-cadherin, E-selectin, ICAM-1 and VCAM-1 in the control and patient groups are shown in Figure 1 (A–D) and Table 2. Elevated soluble adhesion molecule levels were defined as being greater than the 95th percentile of healthy subjects.

© Cancer Research Campaign 1998

**Figure 1 (A–D)** Serum concentration of soluble E-cadherin (**A**), E-selectin (**B**), ICAM-1 (**C**) and VCAM-1 (**D**) in normal healthy controls and patients with colorectal cancer. The median values for each group are shown by horizontal bars and dotted lines represent the 95th percentile of the control group

**Table 2** Serum concentrations of E-cadherin, E-selectin, ICAM-1 and VCAM-1 in the control group of healthy subjects

| | Number of samples | Median ($ng\ ml^{-1}$) | Minimum ($ng\ ml^{-1}$) | Maximum ($ng\ ml^{-1}$) | 95th Percentile ($ng\ ml^{-1}$) |
|---|---|---|---|---|---|
| E-cadherin | 30 | 3.53 | 1.32 | 6.88 | 6.85 |
| E-selectin | 52 | 40.5 | 18.0 | 97.0 | 77.0 |
| ICAM-1 | 52 | 245.5 | 168.0 | 430.0 | 395.8 |
| VCAM-1 | 52 | 695.0 | 451.5 | 1124.2 | 1029.0 |

**Table 3** Spearman rank correlation coefficients for levels of circulating soluble adhesion molecules

| | E-cadherin | E-selectin | ICAM-1 | VCAM-1 |
|---|---|---|---|---|
| E-cadherin | – | | | |
| E-selectin | 0.12 | – | | |
| ICAM-1 | 0.15 | 0.41** | – | |
| VCAM-1 | 0.003 | 0.05 | 0.28* | – |

*$P < 0.05$, **$P < 0.01$.

Soluble ICAM-1 and VCAM-1 concentrations were found to be significantly elevated in comparison with the healthy subjects (median values 349.0 ng $ml^{-1}$, range 160.0–1347.0 ng $ml^{-1}$, $P < 0.00001$ and 986.0 ng $ml^{-1}$, range 426.0–2291.0 ng $ml^{-1}$, $P < 0.00001$ respectively). The concentrations of soluble E-cadherin and E-selectin were not significantly elevated in colorectal cancer patients compared with the control group (median values 3.17 ng $ml^{-1}$, range 1.37–13.04 ng $ml^{-1}$, $P = 0.71$ and 51.0 ng $ml^{-1}$, range 18.0–105.0 ng $ml^{-1}$, $P = 0.052$ respectively), although some patients had high concentrations of the adhesion molecules and with E-selectin the difference between the groups approached statistical significance.

When comparing separately the patients with local and metastatic disease with the control group of healthy subjects, we found significant elevation of concentrations of ICAM-1 and VCAM-1 in both metastatic and non-metastatic colorectal cancer but no increase in levels of soluble E-cadherin and E-selectin regardless of the stage of disease (Figure 2A–D). There was no significant difference between the median serum concentrations of E-selectin, ICAM-1 and VCAM-1 of the patients with local disease (Dukes' stages A and B) compared with the patients with

© Cancer Research Campaign 1998 

**Figure 2 (A–D)** Serum concentration of soluble E-cadherin (**A**), E-selectin (**B**), ICAM-1 (**C**) and VCAM-1 (**D**) in normal healthy controls and patients with colorectal cancer divided into local disease confined to bowel wall (Dukes' A and B) and advanced lymph nodal or metastatic disease (Dukes' C and D). The median values for each group are shown by horizontal bars and dotted lines represent the 95th percentile of the control group

**Table 4** Spearman rank correlation for concentrations of circulating soluble adhesion molecules and clinical, laboratory and pathological parameters

| | E-cadherin | E-selectin | ICAM-1 | VCAM-1 |
|---|---|---|---|---|
| Age | 0.17 | –0.08 | 0.15 | 0.58*** |
| Sex | 0.06 | 0.32* | 0.06 | 0.10 |
| Stage | 0.40* | –0.08 | 0.18 | –0.12 |
| Differentiation | 0.11 | 0.12 | 0.23 | 0.36* |
| Inflammation | 0.28 | 0.02 | 0.10 | 0.19 |
| Ulceration | 0.11 | 0.15 | 0.22 | 0.15 |
| CEA | 0.18 | 0.10 | 0.10 | 0.10 |
| ALT | 0.20 | 0.006 | 0.11 | 0.02 |
| ALP | 0.05 | 0.42** | 0.45** | 0.06 |
| Bilirubin | –0.11 | 0.21 | 0.08 | 0.27 |
| Serum albumin | 0.15 | –0.14 | –0.006 | 0.20 |
| Creatinine | 0.21 | –0.004 | 0.002 | –0.03 |
| Haemoglobin | –0.06 | –0.02 | –0.19 | –0.30* |
| White cell count | 0.34* | 0.49*** | 0.37** | 0.02 |
| Platelet count | 0.21 | 0.30* | 0.41** | –0.10 |

$^{*}P < 0.05$, $^{**}P < 0.01$, $^{***}P < 0.001$.

© Cancer Research Campaign 1998

disease metastatic to regional lymph nodes or liver (Dukes' stages C and D). The patients with Dukes' stages C and D cancer had higher serum concentrations of soluble E-cadherin in comparison with the patients with Dukes' stages A and B but the difference failed to reach statistical significance ($P = 0.051$).

The levels of circulating ICAM-1 were found to be significantly correlated with the levels of circulating VCAM-1 and E-selectin (Table 3).

To determine whether the concentration of soluble adhesion molecules is influenced by tumour cell pathology, presence of inflammation or impaired liver or renal function, the level of correlation between concentrations of soluble adhesion molecules and tumour pathology, stage, markers of liver function (bilirubin, ALP, ALT, serum albumin), renal function (serum creatinine) and routine haematological parameters (haemoglobin, total white cell count and platelet count) was examined (Table 4). The serum concentration of VCAM-1 was significantly correlated with the degree of tumour differentiation with higher serum levels detected in patients with poorly differentiated tumours. No significant correlation was found between the tumour pathology and levels of the other soluble adhesion molecules. However, the analysis was biased by the missing data on grading of differentiation, inflammation and ulceration, with five of the cases being inoperable and five cases being unavailable for independent histological review. When the serum concentrations of adhesion molecules in the group with missing pathology (half of which by definition were more likely to be those with more advanced and unresectable disease) were examined, they were similar to the high-grade tumours, with a trend to be higher than concentrations for low-grade tumours, but the difference did not reach statistical significance (data not shown).

A significant correlation was found between the serum concentrations of E-selectin and ICAM-1 and alkaline phosphatase, total white cell count and platelet count. VCAM-1 was negatively correlated with haemoglobin concentration and positively with age (Table 4). However, no correlation between VCAM-1 and age was observed in the healthy control group, although a slightly young group overall (Spearman rank correlation coefficient = 0.09).

Soluble E-selectin concentrations were higher in men with colorectal cancer than in women (median value for men 61.0 ng $ml^{-1}$, range 18.0–105.0 ng $ml^{-1}$ and median value for women 45.0 ng $ml^{-1}$, range 20.0–81.0 ng $ml^{-1}$, $P = 0.026$), but no association between soluble E-selectin and gender was found in the control group.

As elevation of soluble adhesion molecules has been described in a variety of inflammatory conditions, we collected information on any concomitant illnesses of the colorectal cancer patients (Table 1). Two patients had inflammatory bowel disease, one with diverticulitis and one with an acute gastric ulcer. Elevated serum concentrations of ICAM-1 were observed in all four of them, of E-selectin in three and of VCAM-1 in one. One patient died immediately post-operatively from septicaemia. The soluble adhesion molecule concentrations were normal in this case with the exception of VCAM-1, which was mildly elevated.

### DISCUSSION

This study demonstrates that the serum concentrations of ICAM-1 and VCAM-1 are elevated in patients with colorectal cancer before treatment. The soluble adhesion molecules were elevated both in local and in metastatic disease and no correlation of their concentrations with the stage of the disease was seen. Circulating ICAM-1 has been reported to be increased in malignant melanoma, Hodgkin's disease and gastrointestinal malignancies and has been found to be associated with disease stage and prognosis in melanoma, hepatocellular carcinoma and Hodgkin's disease (Banks et al, 1993; Gruss et al, 1993; Kageshita et al, 1993; Shimizu et al, 1995). Our results extend these observations for patients with colorectal cancer and for the first time report elevated serum concentrations of VCAM-1 in colorectal cancer. This finding is in keeping with our previous observations of elevated VCAM-1 in patients with different gastrointestinal cancers and with gastric cancer, in particular, in which VCAM-1 concentrations seems to have independent prognostic significance (Banks et al, 1993; Velikova et al, 1997).

The source, the molecular nature and the biological significance of the released soluble adhesion molecules is not yet known. VCAM-1 is known to be expressed predominantly on activated endothelial cells, dendritic cells and renal proximal tubule cells. ICAM-1 is expressed on leucocytes, endothelial cells and antigen-presenting cells. Both soluble adhesion molecules are up-regulated by inflammatory cytokines, such as interleukin 1, tumour necrosis factor alpha and interferon gamma (Zetter, 1993). Enzymatic cleavage of cell surface adhesion molecules or secretion of alternatively spliced forms lacking the transmembrane domain have been suggested as possible mechanisms of their elevation in the sera (Meager et al, 1996). Cytokine stimulation of cultured human hepatocytes certainly induces cell surface expression of ICAM-1 and increases soluble ICAM-1 in the culture medium (Thomson et al, 1994). ICAM-1 has been described on malignant epithelial tissue, including metastatic gastric cancer cells, melanoma cell lines and hepatocellular cancer cells (Natali et al, 1990; Koyama et al, 1992; Torii et al, 1993) and may be the source of at least some of the soluble ICAM-1 present in the sera of such cancer patients. However, there are no data describing the expression of ICAM-1 or VCAM-1 on colon cancer cells. Increased expression of E-selectin, ICAM-1 and VCAM-1 has been found in venules around colon cancer primary and metastatic sites (Suzuki et al, 1995), and conceivably these may be the producing sites of circulating adhesion molecules in colon cancer.

After observations of a strong correlation of circulating ICAM-1, VCAM-1, E-cadherin and E-selectin with serum alkaline phosphatase in patients with gastric cancer, we have suggested that soluble adhesion molecules might have a biliary route of excretion and their clearance might be impaired in the presence of intrahepatic cholestasis secondary to liver metastases (Velikova et al, 1997). In the colorectal cancer patients, the correlation between some soluble adhesion molecules and serum alkaline phosphatase is still present but not as strongly as in the gastric cancer patients. This could be due to the higher proportion of patients with advanced and metastatic disease in the group of patients with gastric cancer.

We found that the serum concentrations of ICAM-1 and E-selectin correlated with the total white cell count, suggesting the possibility of release of activating inflammatory cytokines by the white cells up-regulating the release of soluble adhesion molecules from endothelial cells (Carlos and Harlan, 1994; McEver, 1994). Of 13 patients with elevated soluble ICAM-1, only three patients had a white cell count above $10 \times 10^9$ $l^{-1}$, whereas of the four patients with total white cell count above $10 \times 10^9$ $l^{-1}$, soluble ICAM-1 concentrations were outside the normal range in three patients and almost abnormal in the fourth one. This observation

© Cancer Research Campaign 1998

(although based on small numbers) is in keeping with the report of elevated serum ICAM-1 in inflammatory diseases and suggests that release of inflammatory cytokines from white cells is one possible mechanism of elevation of ICAM-1 in patients with colon cancer. It may also suggest a possible role of the soluble adhesion molecules in the inflammatory response of the host. The descriptive information from our study showing an increase in soluble ICAM-1 in all four patients with known inflammatory diseases of the gastrointestinal system emphasizes the probable multifactorial mechanisms of elevation of soluble adhesion molecules and underlines the importance of considering concurrent inflammatory conditions when interpreting measurements of soluble adhesion molecules.

The association of E-selectin and ICAM-1 with the platelet count is interesting in the light of the reported possible endothelial cell activation factor(s) released from activated platelets. Hakomori et al (1994) have suggested that tumour cells can not activate endothelial cells directly but can activate native platelets, which in turn activate endothelial cells to express E-selectin, leading to tumour cell adhesion.

We further observed a positive correlation between serum concentrations of VCAM-1 and the age of the patients. No such correlation was found in the control group of healthy subjects, but we should note that the median age was significantly lower than that of the colorectal cancer patients. Therefore, the possibility of age effect on the serum levels of VCAM-1 can not be excluded, and future studies should take this possible effect into account.

Most studies of soluble adhesion molecules in gastrointestinal malignancies have concentrated on measurements of E-cadherin and E-selectin. Reduced expression of E-cadherin on colon and gastric cancer cells have been associated with dedifferentiation and distant metastasis (Dorudi et al, 1993; Mayer et al, 1993; Shino et al, 1995). Katayama et al (1994) have found significantly increased concentrations of circulating E-cadherin in patients with gastric cancer before surgery. Our study did not confirm these observations in colorectal cancer patients. We did not show any significant increase in serum concentration of E-cadherin or any correlation of soluble E-cadherin levels with degree of tumour differentiation.

E-selectin has been implicated in the adhesion of colorectal cancer cells expressing sialyl Lewis$^x$ and sialyl Lewis$^a$ to activated endothelial cells (Sawada et al, 1994; Izumi et al, 1995; Tozeren et al, 1995). Recent observations have shown that soluble E-selectin and VCAM-1 promote angiogenesis in rat cornea (Koch et al, 1995). Increased serum concentrations of E-selectin have been found in patients with metastatic colorectal cancer but not in patients with non-metastatic disease (Wittig et al, 1996). As the authors have not observed correlation of soluble E-selectin concentrations and markers of inflammation (serum level of C-reactive protein, fibrinogen and tumour necrosis factor alpha), they have proposed that increased soluble E-selectin might reflect neovascularization at the sites of the metastases, linking together the processes of cell adhesion and angiogenesis. We could not confirm the above finding, although in our study a few patients with colorectal cancer had serum concentrations of E-selectin above the normal range, and the difference from the normal group was approaching statistical significance. Possible explanations for the different results in similar groups of patients include: (1) different assays measuring different soluble fragments of E-selectin molecule; (2) relatively small proportion of patients with metastatic disease (11 out of 48) in our patient group and a relatively high proportion of patients with localized colon cancer (28 out of 48). However, these results should encourage further studies looking at serum concentrations of E-selectin in large groups of colorectal cancer patients together with measurement of its known ligand sialyl Lewis$^a$ (CA 19-9), which is a tumour marker test now available for routine clinical practice.

Our study has shown marked elevation of soluble adhesion molecules ICAM-1 and VCAM-1 in patients with different stages of colorectal cancer. Other clinicopathological studies have reported an increase in the serum concentrations of several soluble adhesion molecules, i.e. ICAM-1, E-selectin and more recently VCAM-1, in cancer patients with a range of malignancies and have shown associations with the tumour differentiation, stage and prognosis. A number of in vitro laboratory studies have suggested that adhesion molecules may play a role in the processes of adhesion of tumour cells to endothelium, neovascularization at the metastatic sites and host inflammatory response to cancer. It will be necessary to determine the biological implications and the clinical significance of the many soluble adhesion molecules in cancer.

### ACKNOWLEDGEMENTS

GV, REB, MAF, MJ, JA-M, NRH, PJS are grateful to the Imperial Cancer Research Fund for financial support.

### REFERENCES

Albelda SM (1993) Role of integrins and other cell adhesion molecules in tumor progression and metastasis. *Lab Invest* **68**: 4–17

Bagshawe KD and Rustin GJS (1995) Circulating tumour markers. In *Oxford Textbook of Oncology*, Peckham M, Pinedo H and Veronesi U. (eds), pp. 412–420. Oxford University Press: Oxford

Banks RE, Gearing AJH, Hemingway IK, Norfolk DR, Perren TJ and Selby PJ (1993) Circulating intercellular adhesion molecule-1 (ICAM-1), E-selectin and vascular cell adhesion molecule-1 (VCAM-1) in human malignancies. *Br J Cancer* **68**: 122–124

Banner BF, Savas L and Woda BA (1995) Expression of adhesion molecules in the host response to colon carcinoma. *Ultrastruct Pathol* **19**: 113–118

Carlos TM and Harlan JM (1994) Leukocyte–endothelial adhesion molecules. *Blood* **84**: 2068–2101

Dorudi S, Sheffield JP, Poulson R, Northower JMA and Hart IR (1993) E-cadherin expression in colorectal cancer. *Am J Pathol* **142**: 981–986

Gearing AJH, Hemingway I, Pigott R, Hughes J, Rees AJ and Cashman SJ (1992) Soluble forms of vascular adhesion molecules, E-selectin, ICAM-1 and VCAM-1: pathological significance. *Ann NY Acad Sci* **667**: 324–331

Gruss H, Doelken G, Brach MA, Mertelsmann R and Herrmann F (1993) Serum levels of circulating ICAM-1 are increased in Hodgkin's disease. *Leukemia* **7**: 1245–1249

Hakomori S (1994) Novel endothelial cell activation factor(s) released from activated platelets which induce E-selectin expression and tumour cell adhesion to endothelial cells: a preliminary note. *Biochem Biophys Res Commun* **203**: 1605–1613

Izumi Y, Taniuchi Y, Tsuji T, Smith CW, Nakamori S, Fidler IJ and Irimura T (1995) Characterization of human colon carcinoma variant cells selected for sialyl Lex carbohydrate antigen: liver colonization and adhesion to vascular endothelial cells. *Exp Cell Res* **216**: 215–221

Jones SC, Banks RE, Haidar A, Gearing AJH, Hemingway IK, Ibbotson SH, Dixon MF and Axon ATR (1995) Adhesion molecules in inflammatory bowel disease. *Gut* **36**: 724–730

Kageshita T, Yoshii A, Kimura T, Kuriya N, Ono T, Tsujisaki M, Imai K and Ferrone S (1993) Clinical relevance of ICAM-1 expression in primary lesions and serum of patients with malignant melanoma. *Cancer Res* **53**: 4927–4932

Katayama M, Hirai S, Kamihagi K, Nakagawa K, Yasumoto M and Kato I (1994) Soluble E-cadherin fragments increased in circulation of cancer patients. *Br J Cancer* **69**: 580–585

© Cancer Research Campaign 1998

Koch AE, Halloran MM, Haskell CJ, Shah MR and Polverini PJ (1995) Angiogenesis mediated by soluble forms of E-selectin and vascular cell adhesion molecule-1. *Nature* **376**: 517–519

Koyama S, Ebihara T and Fukao K (1992) Expression of intercellular adhesion molecule 1 (ICAM-1) during the development of invasion and/or metastasis of gastric carcinoma. *J Cancer Res Clin Oncol* **118**: 609–614

Liu D, Nigam AK and Lalani E (1993) Transfection of E-cadherin into a human colon carcinoma cell line induces differentiation and inhibits growth in vitro (abstract). *Gut* **34**: 627

Mayer B, Johnson JP, Leitl F, Jauch KW, Heiss MM, Schildberg FW, Birchmeier W and Funke I (1993) E-cadherin expression in primary and metastatic gastric cancer: down-regulation correlates with cellular dedifferentiation and glandular disintegration. *Cancer Res* **53**: 1690–1695

McEver RP (1994) Selectins. *Curr Opin Immunol* **6**: 75–84

Meager A, Bird C and Mire-Sluis A (1996) Assays for measuring soluble cellular adhesion molecules and soluble cytokine receptors. *J Immunol Methods* **191**: 97–112

Nakamori S, Kameyama M, Imaoka S, Furukawa H, Ishikawa O, Sosaki Y, Kabuto T, Iwanaga T, Matsushita Y and Irimura T (1993) Increased expression of sialyl Lewis x antigen correlated with poor survival in patients with colorectal carcinoma: clinicopathological and immunohistochemical study. *Cancer Res* **53**: 3632–3637

Natali P, Nicotra MR, Cavaliere E, Bigotti A, Romano G, Temponi M and Ferrone S (1990) Differential expression of intercellular adhesion molecule 1 in primary and metastatic melanoma lesions. *Cancer Res* **50**: 1271–1278

Nelson H, Ramsey PS, Donohue JH and Wold LE (1994) Cell adhesion molecule expression within the microvasculature of human colorectal malignancies. *Clin Immunol Immunopathol* **72**: 129–136

Pignatelli M and Vessey CJ (1994) Adhesion molecules: novel molecular tools in tumor pathology. *Hum Pathol* **25**: 849–856

Pignatelli M, Liu D and Nasim MM (1992) Morphoregulatory activities of E-cadherin and beta-1 integrins in colorectal tumor cells. *Br J Cancer* **66**: 629–634

Sawada R, Tsuboi S and Fukuda M (1994) Differential E-selectin-dependent adhesion efficiency in sublines of a human colon cancer exhibiting distinct metastatic potentials. *J Biol Chem* **269**: 1425–1431

Shimizu Y, Minemura M, Tsukishiro T, Kashii Y, Miyamoto M, Nishimori H, Higuchi K and Watanabe A (1995) Serum concentration of intercellular adhesion molecule-1 in patients with hepatocellular carcinoma is a marker of the disease progression and prognosis. *Hepatology* **22**: 525–531

Shino Y, Watanabe A, Yamada Y, Tanase M, Yamada T, Matsuda M, Yamashita J, Tatsumi M, Miwa T and Nakano H (1995) Clinicopathologic evaluation of immunohistochemical E-cadherin expression in human gastric carcinomas. *Cancer* **76**: 2193–2201

Suzuki Y, Ohtani H, Mizoi T, Takeha S, Shiiba K, Matsuno S and Nagura H (1995) Cell adhesion molecule expression by vascular endothelial cells as an immune/inflammatory reaction in human colon carcinoma. *Jpn J Cancer Res* **86**: 585–593

Takeichi M (1993) Cadherins in cancer: implications for invasion and metastasis. *Curr Opin Cell Biol* **5**: 806–811

Thomson AW, Satoh S, Nüssler AK, Tamura K, Woo J, Gavaler J and Van Thiel DH (1994) Circulating intercellular adhesion molecule-1 (ICAM-1) in autoimmune liver disease and evidence for the production of ICAM-1 by cytokine-stimulated human hepatocytes. *Clin Exp Immunol* **95**: 83–90

Torii A, Harada A, Nakao A, Nonami T, Ito M and Takagi H (1993) Expression of intercellular adhesion molecule-1 in hepatocellular carcinoma. *J Surg Oncol* **53**: 239–242

Tozeren A, Kleinman HK, Grant DS, Morales D, Mercurio AM and Byers SW (1995) E-selectin-mediated dynamic interactions of breast- and colon-cancer cells with endothelial cell monolayers. *Int J Cancer* **60**: 426–431

Turnbull RB Jr, Kyle K, Watson FR and Spratt J (1967) Cancer of the colon: the influence of the no-touch isolation technic on survival rates. *Ann Surg* **166**: 420–427

Velikova G, Banks RE, Gearing A, Hemingway I, Forbes MA, Preston SR, Jones M, Wyatt J, Miller K, Ward U, Al-Maskatti J, Singh SM, Ambrose NS, Primrose JN and Selby PJ (1997) Circulating soluble adhesion molecules E-cadherin, E-selectin, intercellular adhesion molecule-1 (ICAM-1) and vascular cell adhesion molecule-1 (VCAM-1) in patients with gastric cancer. *Br J Cancer* **76**: 1398–1404

Wittig BM, Kaulen H, Thees R, Schmitt C, Knolle P, Stock J, Meyer zum Buschenfelde KH and Dippold W (1996) Elevated serum E-selectin in patients with liver metastases of colorectal cancer. *Eur J Cancer* **32A**: 1215–1218

Ye C, Kiriyama K, Mistuoka C, Kannagi R, Ito K, Watanabe T, Kondo K, Akiyama S and Takagi H (1995) Expression of E-selectin on endothelial cells of small veins in human colorectal cancer. *Int J Cancer* **61**: 455–460

Zetter BR (1993) Adhesion molecules in tumor metastasis. *Semin Cancer Biol* **4**: 219–229

© Cancer Research Campaign 1998